# Healthcare for Persons with Intellectual and Developmental Disability in the Community

**DOI:** 10.3389/fpubh.2014.00083

**Published:** 2014-07-15

**Authors:** David A. Ervin, Brian Hennen, Joav Merrick, Mohammed Morad

**Affiliations:** ^1^The Resource Exchange, Colorado Springs, CO, USA; ^2^National Institute of Child Health and Human Development, Jerusalem, Israel; ^3^Department of Family Medicine, Dalhousie University, Dartmouth, NS, Canada; ^4^Health Services, Division for Intellectual and Developmental Disabilities, Ministry of Social Affairs and Social Services, Jerusalem, Israel; ^5^Division of Pediatrics, Hadassah Hebrew University Medical Center, Jerusalem, Israel; ^6^Kentucky Children’s Hospital, University of Kentucky College of Medicine, Lexington, KY, USA; ^7^Center for Healthy Development, School of Public Health, Georgia State University, Atlanta, GA, USA; ^8^Yaski Medical Center, Clalit Health Services, Department of Family Medicine, Faculty of Health Sciences, Ben Gurion University of the Negev, Beer-Sheva, Israel

**Keywords:** community service, primary care, public health, disability, intellectual disability

## Abstract

**Introduction:** While there has been impressive progress in creating and improving community healthcare delivery systems that support people with intellectual and developmental disabilities (IDD), there is much more that can and should be done.

**Methods:** This paper offers a review of healthcare delivery concepts on which new models are being developed, while also establishing an historical context. We review the need for creating fully integrated models of healthcare, and at the same time offer practical considerations that range from specific healthcare delivery system components to the need to expand our approach to training healthcare providers. The models and delivery systems, and the areas of needed focus in their development are reviewed to set a starting point for more and greater work going forward.

**Conclusion:** Today, we celebrate longer life spans of people with IDD, increased attention to the benefits of healthcare that is responsive to their needs, and the development of important healthcare delivery systems that are customized to their needs. We also know that the growing body of research on health status offers incentive to continue developing healthcare structures for people with IDD by training healthcare providers about the needs of people with IDD, by establishing systems of care that integrate acute healthcare with long-term services and support, by developing IDD medicine as a specialty, and by building health promotion and wellness resources to provide people with IDD a set of preventative health supports.

## Introduction

Important steps have been taken over the past decade in addressing the healthcare needs and status of people with intellectual and developmental disabilities (IDD) across their lifespan. People with IDD more fully participate in their communities when they are not constrained by poor health and can access the necessary resources to change conditions affecting their health status (1, 2). Significant barriers remain that prevent greater access to quality healthcare and achievement of desired outcomes. Such barriers include a lack of formal training for healthcare providers, communication deficits between providers and patients, complex and unnecessarily complicated financing systems that limit access to appropriate care, and healthcare providers that lack awareness about steps they might take to ensure that patients with IDD have access to appropriate, culturally competent care (3).

In 2001, 16th US Surgeon General, Dr. David Satcher, published “Closing the gap: a national blueprint to improve the health of persons with mental retardation: report of the Surgeon General’s Conference on Health Disparities and Mental Retardation” (4) in which a priority was made to increase sources of quality healthcare for people with IDD. In 2012, The Arc of the US declared that “all people with intellectual and/or developmental disabilities should have timely access to high quality, comprehensive, accessible, affordable, appropriate healthcare that meets their individual needs, maximizes health, well-being, and function, and increases independence and community participation.”

Progress toward meeting these objectives has been slow, with persistent inequities in health status experienced by people with IDD, and disproportionately higher rates of health problems experienced (5–7). Larson et al. (8) reviewed access and quality issues, concluding that there are “gaps between what individuals with IDD living in community settings need and what they are able to get in health and dental care” (p. 180). The use of emergency departments persist even as people with IDD and healthcare providers acknowledge that emergency departments are inappropriate and more expensive alternatives (9–11) to accessible, quality primary medical care. Predictably, health status among people with IDD continues to lag behind that of the general population.

Systems of care must actively engage people with IDD in health awareness, self-advocacy, health literacy, and health promotion activities to enable them to participate in their own healthcare through improved access (12). People with IDD, their caregivers, and families are often unable to represent their own health concerns due to a lack of understanding of how complex healthcare delivery systems work and not knowing how or in what circumstance to access and employ institutional and community healthcare systems. Healthcare delivery systems must develop and integrate effective networks of primary care medical providers and other health professionals that can positively impact health outcomes for persons with IDD.

## Historical Aspects

Many medical practitioners of the 1950s and 1960s contemplated the health of people with IDD through a disease orientation. In 1954, then President of the American Association on Mental Deficiency (today, the American Association on Intellectual and Developmental Disabilities), Dr. Arthur Hopwood, publicly opined that “medicine, not education, will find the answers” to care and treatment challenges experienced by people with IDD. The person with IDD is, in such a “medical model,” “sick,” which was the basis of institutionalization for decades. Institutions (sometimes called “schools”), which were founded in the late nineteenth and early twentieth centuries on a commitment to educating people with IDD, evolved into centers of custodial care. By the middle of the twentieth century, the educational basis on which most institutions were conceived, had given way to a custodial, administrative model that was negatively referred to as the “medical model” (13). Ironically, people with IDD lived far shorter life spans and experienced far greater health disparities and inequities under the “medical model” than they do today in a community-based model.

In 1962, US President John F. Kennedy (1917–1963), whose sister Rosemary was believed to have an IDD, created the National Institute of Child Health and Human Development within the National Institutes of Health. Kennedy’s efforts also included efforts to deinstitutionalize people with IDD and move them to community, and encouraged the nation’s medical establishment to address the causes and treatment of IDD.

## Deinstitutionalization

Deinstitutionalization has long been viewed as a rejection of the “medical model” of care for people with IDD (14). With large-scale movements of people from institutions during the 1960s and 1970s, it was anticipated that the generic system of health services would be able to provide care for the people with IDD moving into the community. Garrard (15) described the relative absence of critically needed specialty healthcare, including neurology, behavioral neurology, psychiatry, and orthopedic services, needed by people, which “could not be located in the community” (15). Thirty years on, this challenge persists. However, Hayden and Kim (16) found evidence to indicate that people with significant medical conditions can be supported in typical community settings with medical supports found in the community, and that while the community-based healthcare delivery system is in need of improvement, people with IDD who have wide ranges of medical needs can be supported in the community.

## Health Status

As “Closing the gap” outlines, there are a number of reasons for inequities in health status experienced by people with IDD (4). Extensive research identifies obstacles to quality healthcare and attendant health disparities. Poorer outcomes experienced by people with IDD include:

- poorer health with higher rates of preventable mortality, co-morbidities, and chronic conditions; and, less access to preventative care and health promotion;
- inadequacies in mental and oral health services; breast, cervical, and testicular cancer screenings; and immunization updates;
- cognitive challenges in understanding, recognizing, and self-reporting/communicating health problems which affect adherence to treatment;
- financial barriers, even for the insured;
- insufficient healthcare provider incentives to ensure the health of people with IDD;
- mobility/access problems, social and attitudinal barriers, and societal misconceptions;
- lack of research about the healthcare needs of people with IDD; and,
- lack of formal training of healthcare providers, particularly around healthcare needs of adults with IDD, which results in lack of experienced providers in the community.

In 2006, the American Association on Intellectual and Developmental Disabilities (AAIDD) declared “there is a marked disparity of health between persons with IDD and the general population” (17). At about the same time, the United Nations Convention on Rights of Persons with Disabilities was updated to declare the “right [of persons with disabilities] to the enjoyment of the highest attainable standard of health without discrimination on the basis of disability.” Article 25 of the Convention outlines that “persons with disabilities have the right to:

- the same range, quality, and standard of free or affordable health care as provided to other persons;
- health services that are specific to their disabilities, including early identification and intervention as appropriate, and services designed to minimize and prevent further disabilities;
- health services as close as possible to people’s own communities, including in rural areas;
- care of the same quality, including on the basis of free and informed consent by raising awareness of the human rights, dignity, autonomy, and needs of persons with disabilities through training and the promulgation of ethical standards for public and private healthcare; and,
- the provision of health insurance, and life insurance where such insurance is permitted by national law, which shall be provided in a fair and reasonable manner” (18).

**Figure 1:**
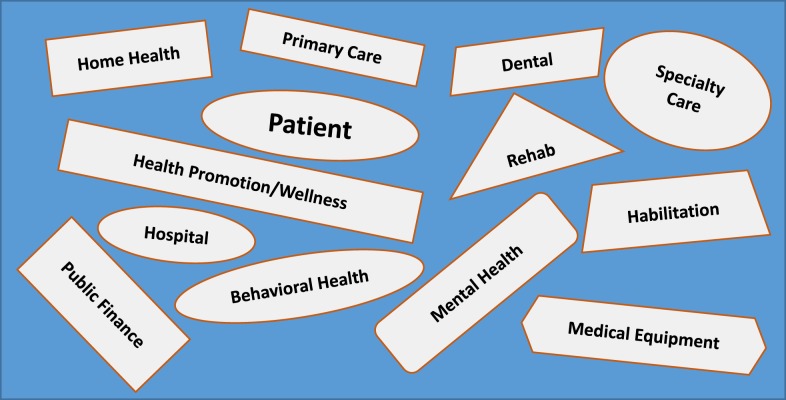
**Healthcare for people with intellectual and developmental disabilities**.

Healthcare for people with IDD is an amalgam of related but distinct component parts, is frequently uncoordinated, and can be extraordinarily difficult to access (see Figure 1). People with IDD are thus confronted with fragmented healthcare in which primary and specialty care is unable to meet their needs (19, 20). There are too few systems in which system components are integrated.

**Figure 2:**
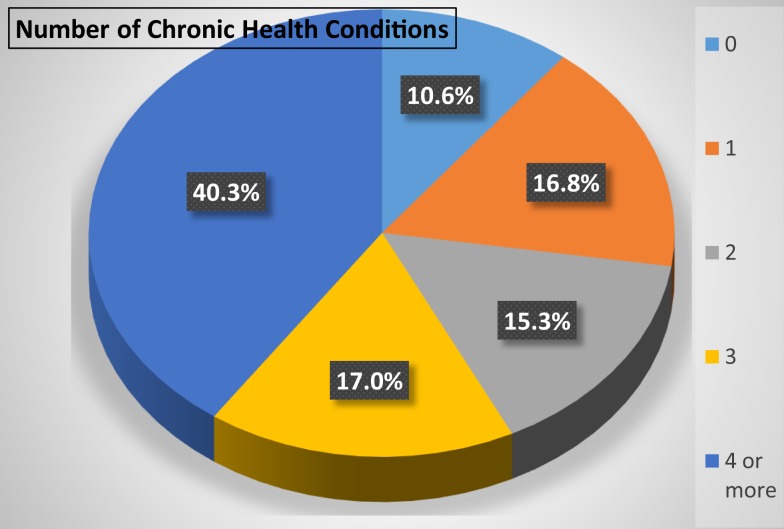
**Number of chronic health conditions in 983 adults with intellectual and developmental disabilities** (23).

This lack of integration is made worse by a range of complicating factors. For example, healthcare providers receive limited training, particularly outside of pediatrics, and thus have limited knowledge of IDD. Nichols et al. (21) noted that most people with IDD receive care from practitioners who have not been appropriately trained and are often unaware of related physical and medical concerns and needs (p. 304). Further, healthcare providers rarely have more than cursory information available to them on the health history of a person with IDD and know little of other aspects of the person’s life (22). The healthcare system focuses on acute medical conditions, not necessarily on related chronic conditions, and is poorly equipped to interact with healthcare needs of people with IDD, who are far more likely to have multiple co-morbidities that are secondary to the IDD. In a 2012 study of 983 adults with IDD, two-thirds had two or more such conditions, with 40.3% having four of more (23) (see Figure 2).

## New Models

The growth of community healthcare delivery systems that offer access to high quality care for people with IDD has been slow but significant. In 2001, “Closing the gap” outlined a number of “programs and creative strategies” from around the US created to address healthcare needs of people with IDD. Among those programs, only two – New York City Premier HealthCare Program and New Jersey’s Developmental Disabilities Health Alliance – were offering community-based, integrated primary care beyond pediatrics. The National Council on Disability’s (NCD) report on “the current state of healthcare for people with disabilities” (3) cited only four “effective programs” delivering healthcare to people with IDD (pp. 241–247).

Other models that have been developed more recently are in early stages of development. In Colorado, for example, the Developmental Disabilities Health Center (DDHC) offers multidisciplinary, fully integrated primary healthcare to adults with IDD. This particular model, conceptualized in 2007 through a meeting (Interagency Action Seminar: building a system of healthcare for adults with developmental disabilities) that brought together local, national, and international experts on healthcare and IDD to develop a model of integrated healthcare customized to the needs of people with IDD, was borne out of five core objectives:

- increase availability of integrated healthcare;
- improve quality of care among community healthcare providers;
- develop health promotion resources for people with IDD;
- increase awareness of healthcare needs among people with IDD; and,
- develop a healthcare delivery system model tailored to the needs of people with IDD.

**Figure 3:**
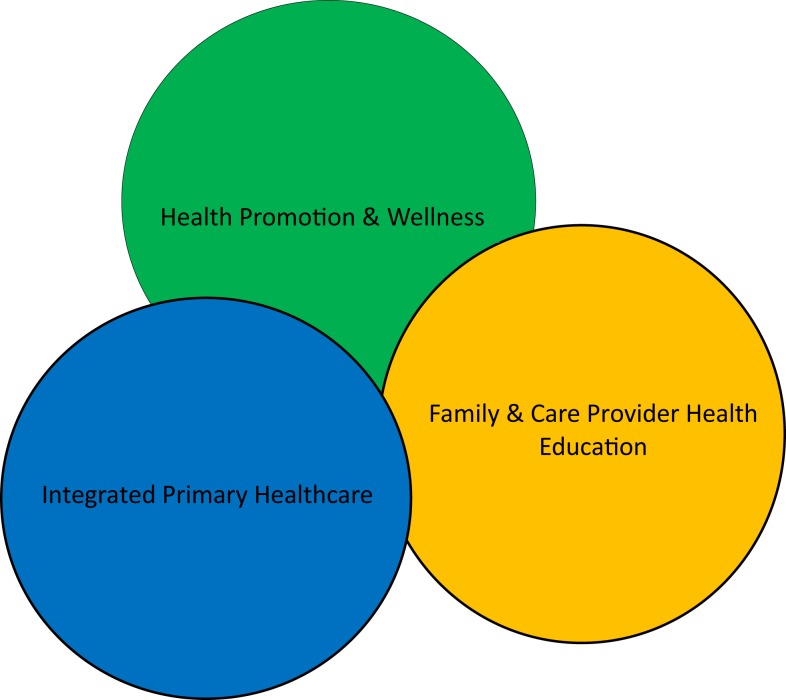
**The major components of healthcare delivery**.

Out of those objectives, a healthcare delivery system concept model was developed (see Figure 3) that is comprised of three major components: primary healthcare, health promotion and wellness, and caregiver education. Within the model, a host of healthcare components and services are included, either directly or by way of formal referral agreements (see Table 1).

**Table 1:** **Healthcare programs and services**.

Customized health promotion programs	Behavioral health services and consultation
Health education programs	Health technologies
Clearinghouse and library of resources	School to adult transition
Integrated mental health	Care coordination
Specialty consults	Primary healthcare
Allied health services	Research and training
Membership networks – family, consumer, providers	Health planning, consultation, and planning

New and related services are being added regularly in response to what people with IDD and their families indicate they need or want.

## Developmental Disabilities Health Center

Opened in 2011, the DDHC in Colorado Springs, CO, USA operates through a partnership of local and national organizations, including a major provider of case management and other traditional developmental disability services, the region’s federally qualified health center (FQHC), the local public hospital, a rehabilitation hospital, and the region’s mental health center.

The DDHC offers integrated primary care that is customized to the needs of children and adults with IDD. This healthcare delivery system is comprised of a number of key elements, including primary care that integrates acute and mental health, behavioral health services and consultation, on-site care coordination, allied health and other specialty care, health planning, and health education programs for patients and their caregivers. The DDHC is also engaged in strategic research and practice partnerships with the University of Colorado, the Coleman Institute on Cognitive Disabilities, the National Center on Health, Physical Activity and Disability (NCHPAD), the Institute on Disability and Human Development (the UCEDD at the University of Illinois at Chicago), the University of Alabama at Birmingham, and the National Institute on Child Health and Human Development in Israel.

## International

It is important to acknowledge differences among countries in how health services are provided to persons with IDD and how healthcare providers learn about IDD. In the UK, Australia, New Zealand, Canada, and the Netherlands, for example, primary medical care to persons with IDD and their families is provided by general practitioners or family physicians and takes place in the community. Consultant specialists see patients on referral and provide most hospital care. Except for children with complex and severe disability and who are near to large hospital centers and specialists, the family physician/general practitioner is the main medical provider.

Developmental pediatricians in large hospital centers provide a large portion of primary care through a range of clinics (e.g., developmental clinic, complex care clinic, genetics clinic, Down syndrome clinic, Autism clinic, etc.). Other specialists, working out of the same facility are accessible (e.g., orthopedics, neurology, psychiatry, dentistry). For adults with IDD, with rare exceptions, there are no such focused resources.

In the US, primary medical care may be provided by family physicians, general pediatricians, general internists, and, for women, even obstetricians/gynecologists. Consulting specialists work out of larger hospital centers, some providing continuing management for particular conditions related to their specialty. Special clinics for IDD are found in children’s hospitals with variable emphases (general developmental clinic, syndrome-specific clinics, genetics clinic, etc.). Again, for adults with IDD, there are few focused resources and the primary care physician, a family physician, or internist, is expected to provide continuing, comprehensive medical care.

While these and other emerging models are excellent examples of innovative approaches to caring for people with IDD, there are few well-designed studies underway to test their impact.

## Provider Training and Education

Medical education undergraduate programs have variable curricular emphasis on IDD. There are few national standards, and what happens in a particular medical school often depends on the presence or absence of faculty “champions” who advocate for IDD content. Recently, in Canada, the national medical exam (Medical Council) has included IDD in both child-focused and adult-focused “learning objectives” as an expectation in undergraduate medical curricula.

In residency or traineeships, whether curricular programs of primary care or consulting specialties, there are no national expectations. One exception is found in the UK where in psychiatry a 3-year program (after already a specialist in psychiatry) will result in the sub-specialty of learning disability (in the UK this is the term for intellectual disability). In Canada, there is one post-residency fellowship in family medicine – developmental disabilities, each year at Queen’s University. In the Netherlands, the only IDD specialty training program anywhere is 3 years long and offered to graduate general practitioners (24).

It is a reality that graduates of medical school anywhere have no accreditation-required curriculum in IDD. It is also the case that, except for pediatrics generally and psychiatry in the UK, no general practice/family medicine or consulting specialty post graduate program offers core curriculum in IDD. Yet in all world countries, persons with IDD look to general practitioners/family physicians for their continuing, comprehensive primary medical care.

Some medical schools are beginning to offer undergraduate curriculum content in IDD, usually found in psychology, psychiatry, or pediatrics programs and are often community-based. In the US, some involved medical schools are Ohio State, University of Iowa, University of the Pacific, University of New Hampshire, Matheny Medical and Education Center, Tufts University School of Medicine, Villanova University, University of South Florida, and University of South Carolina.

## Integration

In healthcare generally, there are many effective integrated models of care. There are, for example, bi-directional referral systems, the least integrated and perhaps more properly named collegial, created out of healthcare provider relationships with other providers who may be located in another part of the same building, across the street, or across town. Location does not drive the connection, but a working relationship with mutual interest in the well-being of the patient drives the referral process. This approach can develop informally and simply arises from both providers’ efforts to develop a referral network. Co-location of health providers within the same building or clinic also emerges as a step toward integrating care. Providers exchange information about the patients they have in common on an as-needed basis. Referrals commonly flow from the physician to the mental health provider when signs of need for mental or behavioral health supports are noted during a primary care visit. More fully integrated care includes having both the mental health provider and physician in the same room with the client at the same time, a co-visitation model. The efficacy of this approach is well-documented (25, 26).

**Figure 4:**
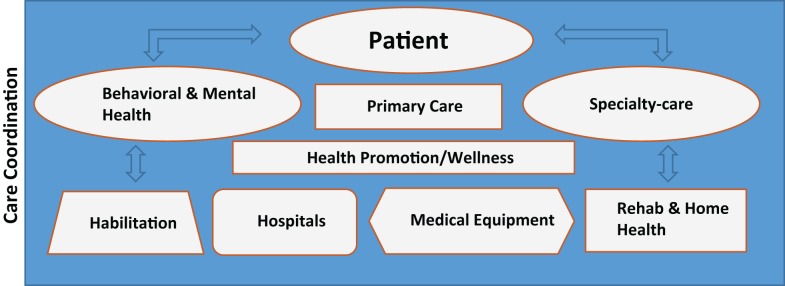
**Integration of care**.

As our understanding of the health needs and experiences of people with IDD advances, we find value in integrating not just the many potential elements of acute healthcare, but also in linking acute with behavioral health, long-term service and support systems, and the community-based social and developmental support structures of the person with IDD (Figure 4).

It is important to note that while a person may access all of these elements, it is only when all are fully integrated that optimal health becomes achievable. This approach emphasizes the need for careful care coordination, beyond traditional healthcare models, to include all major aspects of a person’s life.

## Future Aspirations

In its joint position statement on health, mental health, vision, and dental care (as adopted in February 2013), the AAIDD and the Arc of the US noted that “while many people encounter difficulty in finding affordable, high-quality healthcare, people with IDD face additional barriers, sometimes life-threatening, when attempting to access timely, appropriate health services in their communities,” including:

- Access – underinvestment in public health and wellness to people with IDD results in preventable healthcare disparities and poorer health outcomes. Inadequate training, lack of coordinated care, and inadequate levels of reimbursement are some of the programmatic barriers while inaccessible clinical settings and diagnostic and medical equipment, along with translation and interpretation challenges, create physical barriers.
- Discrimination – healthcare providers sometimes provide inadequate or inappropriate interventions and treatments or deny appropriate care for people with IDD because of professional ignorance as well as personal and/or societal bias. State statutory liability damage limits discriminate against people with severe and/or life-long disabilities because they fail to provide sufficient compensation.
- Affordability – people with IDD are more likely to live in poverty and cannot afford cost-sharing. To save costs, many public and private health care plans limit access to specialists and critical services. Services are often available in a community, but many people with IDD lack adequate public or private insurance to pay for them.
- Communication and personal decision-making – people with IDD may have difficulties communicating their needs and making healthcare decisions without support. Their decisions, even those made with support of a surrogate decision-maker may not be respected and implemented by healthcare providers.

Important steps have been made toward the removal of such barriers: the development of a number of integrated healthcare delivery systems, an increasing number of books and journals that together are forming a portfolio of best practice guidelines, and the development of recognized specialties in IDD medicine abroad. Such steps lead to greater recognition of the healthcare needs of people with IDD, and the appropriate systems of care needed to address them. Through accumulated experience and analyses of health status of people with IDD, we are learning important lessons that can contribute to our body of knowledge and inform the continued development and evolution of healthcare delivery systems.

## Healthcare Delivery Systems

For some time, healthcare delivery systems that integrate a range of services have been seen as key to achieving good health outcomes and important financial efficiencies. These integrated delivery systems are collaborative networks that link healthcare providers in a coordinated, vertical continuum of services to a particular patient population or community (27). There are practical considerations in the development of these delivery systems that need attention. For example, in developing Colorado’s DDHC, these items were considered critical:

- appointments are lengthened, typically set for as long as 60 min to allow the healthcare provider adequate time to establish a rapport and relationship with the patient.
- Lighting is natural and non-fluorescent to accommodate patients with sensory integration issues (28, 29).
- Examination room size was enlarged (up to 224 ft^2^, 21 m^2^) to accommodate wheelchairs and other adaptive equipment, and to allow the patient to have as many people accompany them as they need to feel safe and comfortable.
- Equipment choices include a range of specialized equipment, from “high/low” examination tables, to four different types of scales, including wheelchair and “grab bar” scales; and, comfortable and welcoming decor. The clinic was designed by, with, and for people with IDD.

## Standards of Care

Sullivan et al. (30) highlighted that primary care providers are often the most consistently available health professionals involved in caring for people with IDD and in interacting with regular caregivers. Their contribution is vital for disease prevention, early detection, and appropriate management. They can help to assess the need for referral to specialized and interdisciplinary health services when these are available. They also provide continuity and coordination of care. Reliable guidelines, however, are required to inform primary care providers about the particular health needs of people with IDD and the best approaches to management.

As long ago as 2003, the Dutch Society of Physicians for Persons with Intellectual Disabilities began formulating basic standards of care for people with IDD. The resulting manifesto “basic standards of healthcare for people with intellectual disabilities (31),” outlines five criteria for healthcare for people with IDD: that people with IDD will use mainstream health services, that health professionals will have competencies in IDD, that health professionals who are specialized in the specific health needs of individuals with IDD are available as back-up to mainstream service providers, that a multidisciplinary approach is indicated, and that a proactive, preventive, and anticipatory emphasis be placed on the delivery of healthcare for people with IDD.

In Canada, the Developmental Disability Primary Care Initiative has resulted in an emerging set of peer-reviewed guidelines and tools to guide healthcare delivery to people with IDD (32). Accreditation Canada, a non-profit organization that accredits healthcare and social services, in 2010 established a set of standards “providing services for people with developmental disabilities” (www.accreditation.ca).

These promising approaches to establishing standards of care are important steps; however, more needs to be done to expand them globally. We highlight Horwitz et al. (33), who 14 years ago, recommended that guidelines be established to ensure the quality of care and raise healthcare provider competence and confidence in providing appropriate, responsive healthcare to people with IDD. Such guidelines should be developed with substantial input from people with IDD and their families, physicians and other healthcare providers, researchers, and other healthcare delivery system stakeholders.

## Healthcare Financing

In “Closing the gap,” one of six core objectives is a systematic assessment of healthcare financing. That healthcare financing is complex and unnecessarily complicated is apparently the only point on which everyone can agree. In the US, healthcare spending and the health status of Americans are not correlated. From private and public funding sources, payments are made in relation to particular procedures rather than as incentives for health outcomes. For people with IDD in the US, who rely heavily on publicly financed healthcare (Medicaid and Medicare), the situation is exacerbated by below-market reimbursements to healthcare providers, which has resulted in fewer and fewer providers who will accept Medicaid and Medicare patients. This leaves people with IDD with a dangerously constricting set of quality healthcare options.

Bersani and Lyman (34) offer a thorough outline of US government programs that support people with disabilities and particularly on the programs available to support access to healthcare (pp. 98–102). A core element of these programs is Medicaid, a federally funded, state-managed system that pays for healthcare on a fee-for-service basis. People with IDD are, in the vast majority, eligible for and widely enrolled in Medicaid.

In other countries, healthcare financing systems vary. In the UK, Canada, Israel, and most other industrialized nations, equal access to healthcare is assured through government-controlled universal coverage health services. While this assures access, quality concerns remain. Private insurances, either as primary funders of healthcare services (as in the US) or as a supplement to universal coverage schemes (as in Israel, for example), are not a guarantee of access for people with IDD nor are they correlated to the quality of the healthcare provided or outcomes achieved. In the last decade, laws have appeared in a number of US states to mandate minimum coverage under private insurance plans that offer greater access to healthcare services for people – particularly children – with IDD. This has resulted in mandatory private insurance coverage, for example, of therapies such as applied behavior analysis for children with Autism. Furthermore, the US Patient Protection and Affordable Care Act requires that individual and small private insurance group plans cover “rehabilitative and habilitative services and devices,” benefits that have previously not been widely available under private insurance plans. These important expansions of covered services through public and private insurance are steps in the right direction for people with IDD and their families.

## Conclusion

In the mid-1970s, the American Association on Mental Deficiency declared that the presence of an intellectual disability “is no justification for permitting any human life to be terminated through the withholding of life-sustaining procedures.” The need for such a statement at all implies what we know historically, that people with IDD experience extremely limited access to quality healthcare. Today, we celebrate longer life spans of people with IDD, increased attention to the benefits of healthcare that is responsive to their needs, and the development of important healthcare delivery systems that are customized to their needs. We also know that the growing body of research on health status offers incentive to continue developing healthcare structures for people with IDD by training healthcare providers about the needs of people with IDD, by establishing systems of care that integrate acute healthcare with long-term services and support, by developing IDD medicine as a specialty, and by building health promotion and wellness resources to provide people with IDD a set of preventative health supports that did not exist 25 years ago. These and other important advancements in our understanding of the health status and healthcare needs of people across the lifespan can only be characterized, against the backdrop of the realities for people with IDD in the mid-twentieth century, as extraordinary.

There is more still to be done.

- Standards of care for people with IDD and their families need to be expanded and codified. These standards should account for the unique healthcare needs of people with IDD, should integrate concepts of self-direction and self-determination, and should reflect the need for and benefits of integration. End-of-life and palliative care issues need to be addressed also.
- Training and education for healthcare providers needs to be expanded globally. Particularly in the US, but also in other parts of the world, medical schools need to develop and formalize training that emphasizes communication skills and clinical experience, and create residencies and post-residency fellowships in IDD medicine. Other health professions’ educational programs similarly require developmental disabilities to be strengthened in their curricula.
- Board certification should be created and available for physicians who seek specialization in IDD medicine. While specialties in developmental and neurodevelopmental pediatrics are recognized, there are virtually no options for equivalent adult-directed specialty development.
- Healthcare delivery systems, building on existing models as well as innovating new approaches to addressing the healthcare needs of people with IDD, need to be developed to improve access to quality care. As recently as 5 years ago, the NCD published four projects noted as “effective programs” delivering healthcare to people with developmental disabilities. More replicable models and research on their efficacy is needed.
- Health promotion, wellness, and disease prevention strategies addressing health issues that are unique to people with IDD need to be strengthened. Important work has been done in this area (35–37). Projects like the NCHPAD in the US, and the Rehabilitation Research and Training Center on Developmental Disabilities and Health (RRTCDD) at the University of Illinois at Chicago are examples of what is essential to develop our understanding of health promotion.
- Research on the relationships between health status and quality of life, on systems of healthcare delivery and health status, and on the benefits of health promotion and disease prevention on health status needs to be expanded. While there is sufficient research to conclude that people with IDD experience health disparities, research should examine interventions at both the clinical treatment level and the policy development level.

By building on the recent past successes, and by attending to these recommendations and other needed advancements in our approach to healthcare for people with IDD, we can be assured of improving the access to quality healthcare for all people.

## Conflict of Interest Statement

The authors declare that the research was conducted in the absence of any commercial or financial relationships that could be construed as a potential conflict of interest.
